# Aqueous extract of *Cordyceps cicadae* (Miq.) promotes hyaluronan synthesis in human skin fibroblasts: A potential moisturizing and anti-aging ingredient

**DOI:** 10.1371/journal.pone.0274479

**Published:** 2023-07-07

**Authors:** Li Shao, Sujing Jiang, Yan Li, Ling Yu, Hui Liu, Laiji Ma, Suzhen Yang

**Affiliations:** 1 School of Perfume and Aroma Technology, Shanghai Institute of Technology, Shanghai, China; 2 R&D Innovation Center, Shandong Freda Biotech Co., Ltd, Jinan, China; Indian Institute of Technology Delhi, INDIA

## Abstract

*Cordyceps cicadae* (Miq.) is an edible fungus with unique and valuable medicinal properties that is commonly used in traditional Chinese medicine, but its anti-aging effects on the skin fibroblast are not well studied. The aim of the present study was to analyze the active components of aqueous *C*. *cicadae* extract (CCE), determine the effects of CCE on hyaluronan synthesis in human skin fibroblasts, and explore the underlying mechanisms. The results of this study indicate that CCE was rich in polysaccharides, five alditols (mainly mannitol), eight nucleosides, protein, and polyphenols, which were present at concentrations of 62.7, 110, 8.26, 35.7, and 3.8 mg/g, respectively. The concentration of extract required to inhibit 50% of 2,2-azino-bis (3-ethylbenzothiazo-line-6-sulphonic acid) (ABTS) and 2,2-diphenyl-1-picrylhydrazil (DPPH) radical scavenging capacities were 0.36 ± 0.03 and 4.54 ± 0.10 mg/mL, respectively, indicating that CCE exhibits excellent antioxidant activities. CCE showed no cytotoxicity to skin fibroblasts at concentrations ≤ 100 μg/mL, and promoted HA synthesis in fibroblasts. Treatment of fibroblast cells with 100 μg/mL CCE enhances the HA content to 1293 ± 142 ng/mL, which is significantly more than that in the non-treatment (NT) group (p = 0.0067). Further, RNA sequencing detected 1,192 differentially expressed genes (DEGs) in CCE-treated fibroblasts, among which 417 were upregulated and 775 were downregulated. Kyoto Encyclopedia of Genes (KEGG) and Genomes pathway (GO) analysis based on RNA sequencing revealed that CCE mainly affected cytokine-cytokine receptor interaction regulated by HA synthesis-related genes. CCE upregulated HA synthase 2 (*HAS2*), epidermal growth factor (EGF)-related genes, heparin-binding EGF-like growth factor, C-C motif chemokine ligand 2, interleukin 1 receptor-associated kinase 2, and other genes related to fibroblast differentiation and proliferation. CCE downregulated the gene of matrix metallopeptidase 12 (*MMP12*), which leads to cell matrix loss. RT-qPCR further verified CCE significantly upregulated *HAS2* expression and significantly downregulated *MMP12* expression, thus promoting hyaluronan synthesis. CCE shows potential as a moisturizer and anti-aging agent in functional foods and cosmetics.

## 1. Introduction

*Cordyceps cicadae* (Miq.) is a medicinal fungus that parasitizes Hemiptera cicadae larvae [1]. *C*. *cicadae* is a valued ingredient in Traditional Chinese Medicine (TCM) that has been used for approximately 1,600 years [2]. Active components, such as polysaccharides, nucleosides, sterols, cyclic dipeptides, sugars, fatty acids, amino acids, aromatic compounds, and other small organic compounds, have been isolated from *C*. *cicadae* [3]. Many pharmacological studies have demonstrated that *C*. *cicadae* exhibits biological functions such as growth reduction of human hepatocellular carcinoma cells, prevention of cell senescence, bacteriostasis [2, 4, 5] antioxidant activities [2, 4, 5], prevention of liver damage, anti-fatigue and anti-aging [3, 6, 7].

The skin, which is the largest organ in the mammalian ectoderm, ages with time and external factors [8]. Skin aging is characterized by skin water loss, sagging, increased wrinkles, and decreased elasticity. Histologically, the extracellular matrix (ECM) loses flexibility due to the breakdown of skin collagen and elastic fibers and a reduction in hyaluronan (HA) content [4, 9]. HA is a disaccharide unit glycosaminoglycan composed of D-glucuronic acid and N-acetylglucosamine. It is a natural component in the skin that helps absorb moisture from the body and the skin surface and is particularly prominent in rapidly growing and repairing tissues [10]. In addition, HA is involved in wound healing, ECM tissue and joint lubrication, regulation of cell adhesion and movement through receptors that interact with the cytoskeleton, angiogenesis by mediating cell proliferation, cell differentiation, and cell migration and has immunomodulatory, anti-tumor, and anti-proliferative properties [11–13].

Natural products are rich in biological ingredients known for their therapeutic properties. Recent research on the antioxidant, anti-inflammatory, and antibacterial activities of *Cordyceps* fungus extracts suggests that they have great potential for biomedical and cosmetic applications [14]. *C*. *cicadae* belongs to the *Cordycipitaceae* family, which is known for its therapeutic and health benefits. However, research on the potential skin care applications of *C*. *cicadae* is limited. In this study, we obtained *C*. *cicadae* extract (CCE) by hot water extraction, analyzed its active components, and evaluated its antioxidant properties and effects on HA synthesis in human skin fibroblasts. Further, we determined the effects of CCE on human fibroblasts using transcriptome analysis and verified the expression of HA-related genes using RT-qPCR. This study will further our knowledge of *C*. *cicadae*, its effects on human fibroblasts, and the mechanisms involved in the promotion of HA synthesis.

## 2. Materials and methods

### 2.1 Material

*C*. *cicadae* samples were purchased from Jingfu Traditional Chinese Medicine Decoction Pieces Co., LTD, Bozhou City, batch number 210901. The product had the characteristics of *C*. *cicadae* and met the Shanghai processing specifications of TCM decoction pieces, inspection number JC210901.

### 2.2 Chemicals

1,1-diphenyl-2-picrylhydrazyl (DPPH) was purchased from Sigma-Aldrich; 2,2-azino-bis (3-ethylbenzothiazo-line-6-sulphonic acid) diammonium salt (ABTS), Trolox, and the ferric reducing antioxidant power (FRAP) assay kit were purchased from the Beyotime Institute of Biotechnology (S0119 and S0116, Nantong, China). Dulbecco’s modified eagle medium (DMEM), amphotericin B, penicillin, bovine serum albumin (BSA), and Dulbecco’s phosphate-buffered saline (DPBS) were purchased from Thermo Fisher Scientific Inc. (China); streptomycin was purchased from Guangzhou Huayueruike Scientific Equipment Co. (China); Hyaluronan Quantikine ELISA Kit was purchased from R&D Systems (DHYAL0). All other reagents and solvents were of analytical grade.

### 2.3 *C*. *cicadae* extraction

*C*. *cicadae* fruiting bodies were separated, washed with distilled water, and dried at 60°C until reaching a constant weight. Dried fruiting bodies were ground into powder and passed through an 80 mesh sieve. Ten grams of *C*. *cicadae* powder was dissolved in 200 mL water and heated at 85°C for 2.5 h. The extraction solution was separated by centrifugation at 8,500 × *g* for 15 min and lyophilized to obtain CCE for quantitative assessment and analysis.

### 2.4 Determination of active components

#### 2.4.1 Polysaccharide extraction and assessment

Crude polysaccharides were extracted following the standard method with some modifications [2]. Ten grams of CCE powder was dissolved in 20 mL water, and three times the volume of alcohol was added to the extract solution. The mixture was incubated at 4°C for 12 h, and then the precipitate was separated by centrifugation at 8,000 × *g* for 15 min. The crude polysaccharides precipitate was washed with deionized water and lyophilized for quantitative assessment via the phenol-sulfuric acid method [15]. The linear relationship between the polysaccharide content and the absorbance at 490 nm was determined. Glucose solution was used as the standard.

#### 2.4.2 Monosaccharide composition and molecular weight analysis

The lyophilized polysaccharide sample was weighed, and 2–5 mg were added to a hydrolysis flask with 3 mL of trifluoroacetic acid (4 mol/L). The mixture was hydrolyzed at 110°C for 3 h. After hydrolysis, 3 mL of methanol was added, and nitrogen was used to blow dry. This was repeated three times, and distilled water was used to dissolve the volume to 50–100 mL. The solution was filtered through a 0.45-μm membrane before determination. The filtrate was assessed using high performance anion exchange chromatography (HPAEC) according to the method described by [16]. The chromatography column was a CarboPac PA20 column (3 mm × 150 mm, Dionex, USA) with pulsed amperometric detection. The mobile phase was ultrapure water (A), 0.25-mol/L NaOH (B), and 1-mol/L NaAc (C), with the following elution gradient: 0–30 min: 99.2% A and 0.8% B; 30–40 min: 79.2% A, 0.8% B, and 20% C; 40–45 min: 20% A and 80% B; 45–60 min: 99.2% A and 0.8% B. The flow rate was 0.5 mL/min and the injection volume was 20 μL. Fucose, rhamnose, arabinose, galactose, glucose, xylose, mannose, fructose, galacturonic acid, and galacturonic acid were mixed as a standard.

The molecular weight of polysaccharides in CCE was determined according to previously reported methods with modifications [17]. A TSK-GEL G6000 PWXL chromatographic column (7.8 mm × 300 mm, Tosoh Corp., Tokyo, Japan) with an injection volume of 20 μL was used for the analysis. Ultrapure water elution was conducted at a flow rate of 0.6 mL/min at a column temperature of 30°C.

#### 2.4.3 Protein quantification

Protein content was determined using a Bradford assay [18]. The protein sample (1 mL) was combined with 5 mL of Coomassie Brilliant Blue G-250 solution, mixed, and incubated at room temperature for 20 min. BSA concentrations (0, 0.025, 0.05, 0.1, 0.15, 0.2, and 0.25 μg/mL) were prepared respectively for standards. The absorbance was measured at 595 nm, and the results were reported as milligrams of BSA equivalents per milligram of extract.

#### 2.4.4 Alditols analysis

Alditols contents were determined by HPAEC. CCE powder (0.025 g) was dissolved in 100 mL distilled water and the solution was filtered through a 0.45-μm microporous membrane before ion chromatography determination using a CarboPac MA1 anion exchange column (4 mm × 250 mm, Dionex, USA). The injection volume was 25 μL, the mobile phase was 48-mmol/L NaOH, the flow rate was 0.4 mL/min, and the column temperature was 30°C.

#### 2.4.5 Nucleoside analysis

CCE nucleosides were analyzed by high-performance liquid chromatography (HPLC). CCE powder (0.5 g) was dissolved with 50 mL distilled water, and the solution was filtered through a 0.45-μm aqueous phase filter membrane for liquid chromatography analysis. The chromatography column was a Venusil MP C18 column (5 μm, 4.6 mm × 250 mm, Agela Technologies of America). The mobile phase was composed of ultrapure water (A) and methanol (B) with a gradient elution as follows: 0–5 min: 100% A; 5–10 min A: 95 A; 10–30 min: 70% A; 30–40 min: 95% A; 40–45 min: 100% A. The injection volume was 10 μL, the flow rate was 1.0 mL/min, and the column temperature was 40°C. The UV detection wavelength was set at 254 nm.

#### 2.4.6 Total phenolic content analysis

The total phenolic content in CCE was determined using a Folin-Ciocalteu assay as described by Bursal et al. [19]. CCE dissolved to 1 mg/mL (1 mL) was added to a test tube with 1 mL of Folin-Ciocalteu reagent and 2 mL of 2% Na_2_CO_3_. The reagents were vortexed and incubated at 25°C for 40 min. Absorbance was measured at 765 nm. The results were reported as milligrams of gallic acid equivalents per milligram of extract.

### 2.5 Antioxidant activity assays

#### 2.5.1 ABTS radical scavenging activity assay

ABTS radical scavenging activity was assayed according to instructions from the Beyotime Institute of Biotechnology. ABTS solution was prepared by mixing the stock solution and oxidant solution in equal volumes and allowing them to react at room temperature in the dark for 16 h. A 10-μL aliquot of CCE solution was added to 200 μL of fresh ABTS solution. The solution was then diluted with 5-mM phosphate-buffered saline (pH 7.4) to obtain an absorbance of 0.7 ± 0.05 at 734 nm. The absorbance was measured at 734 nm after 6 min at room temperature. Ascorbic acid was used as a reference; the ABTS radical scavenging activity was calculated using Eq 1.

$$\text{Scavenging}\;\text{activity}\;\left(\%\right)=1-\frac{\left(A_{t}-B\right)}{A_{0}}\times 100$$ (1)

where *A*_*t*_ is the absorbance of the sample, *B* is the absorbance of the sample and distilled water, and *A*_*0*_ is the absorbance of the blank control.

#### 2.5.2 DPPH radical scavenging activity assay

The free radical scavenging activity of CCE was analyzed by a 2, 2-diphenyl-1-picrylhydrazil (DPPH) assay as described by Sharma et al. [15]. First, the CCE solution (0.5, 1, 2, 3 and 4 mg/mL) was prepared by diluting CCE in distilled water and add 0.4 mL solution in the test tubes respectively. Finally, 3.6 mL of DPPH solution (0.1 mM) was transferred to the test tubes, which were gently stirred and incubated in the dark at 25°C for 30 min. The absorbance was read at 517 nm using a spectrophotometer, and the scavenging activity (%) of each concentration was calculated in triplicate (Eq 2). Distilled water was used as the blank control.

$$AA\;(\%)=\;\frac{A_{0}-\left(A_{S}-A_{C}\right)}{A_{0}}\times 100$$ (2)

where *A*_*0*_ is the absorbance of the blank control, *A*_*s*_ is the absorbance of the sample, and *A*_*c*_ is the absorbance of the sample and ethanol mixture.

#### 2.5.3 FRAP assay

The ferric reducing ability of CCE was determined by FRAP assay as per instructions from the Beyotime Institute of Biotechnology. Stock solutions were the detective buffer, 2, 4, 6-tripyridyl-s-triazine (TPTZ) solution, TPTZ dilution, 0.5 mL of 10-mM FeSO_4_ solution, and 0.1 mL of 10-mM Trolox solution. A working solution was prepared by mixing TPTZ dilution, detective buffer, and TPTZ solution in a ratio of 10:1:1 (v/v), respectively. The working solution was warmed to 37°C before use. CCE samples (5 μL) with concentrations ranging from 0.3 to 1.5 mM were mixed with 180 μL of FRAP working solution and incubated at 37°C for 5 min. The absorbance of the reaction mixture was measured at 593 nm. FRAP was expressed as FeSO_4_ concentrations, which were calculated using standard curves.

### 2.6 Effects of CCE on fibroblast HA synthesis

#### 2.6.1 Cell culture

Human skin fibroblasts, which were collected from the facial skin of a 45-year-old woman, were obtained from the Shanghai Engineering Research Center cell bank. The cell culture was prepared in regular DMEM. After resuscitation, fibroblasts were cultured in 175 cm^2^ flasks for 7 days, and the medium was refreshed every other day. The prepared CCE medium was added to each well containing fibroblasts and cultured at 37°C for 24 h. A 5% BSA blocking buffer, which was devolved in high glucose DMEM and DPBS, was used to block the cells to prevent false positives. Cells treated with regular DMEM served as the not treated (NT) group. For cell viability assays, the cells were cultured in 96-well plates at a concentration of 10,000 cells/well and cultured in 6-well plates at a concentration of 20,000 cells/well for immunofluorescence analysis of HA expression.

#### 2.6.2 Cell viability assay

Cell viability was determined using a 3-(4,5-dimethylthiazol-2-yl)-2,5-diphenyl-2H-tetrazolium bromide (MTT) assay [20]. Serum-free medium was used to starve the cells for 12 h, and medium solutions with a CCE concentration gradient of 10 to 1,000 μg/mL CCE were added to the wells, followed by incubation at 37°C for 24 h. Three parallel tests were set for each concentration. The medium was removed after 24 h, and 0.5-g/L MTT solution was added to the wells, followed by incubation at 37°C for 3 h. Dimethylsulfoxide was added, and the absorbance was measured at 550 nm.

#### 2.6.3 Determination of HA content

CCE solutions were prepared to two specific concentrations (50, 100 μg/mL), with regular DMEM (high glucose DMEM with 10% bovine calf serum, penicillin, streptomycin, and amphotericin b) and passed through a 0.22-μm filter. Fibroblasts were starved in a 24-well plate using serum-free medium for 16 h. Subsequently, cells were further treated with CCE (50, 100 μg/mL) at 37°C for 48 h. Cell supernatants were collected and assayed for HA content according to the guidelines of the ELISA kit (R&D systems, Cat. Number: DHYAL0) [21].

#### 2.6.4 Immunofluorescence analysis of HA expression

Sheep anti-HA antibody was diluted with BSA (1:500) to prepare the antibody-BSA working solution. One milliliter of the antibody-BSA working solution was added to each well and incubated at 4°C for 24 h. The antibody-BSA working solution was removed from each well, and the wells were rinsed with 3 mL of DPBS twice. Goat anti-sheep antibody 488 and 4′,6-diamidino-2-phenylindole fluorescent stain were diluted in 5% BSA blocking buffer (1:500 and 1:1,000, respectively) to prepare the anti-sheep 488 working solution. One milliliter of the working solution was added to each well and incubated at 4°C for 1 h. Next, the antibody-BSA working solution was removed from each well, and the wells were rinsed with 3 mL DPBS twice. Three pictures of each well were taken at random using an A1 fluorescence microscope (Zeiss Group, Germany).

### 2.7 Transcriptome sequencing

Total RNA was extracted from fibroblasts using the RNeasy mini kit (Qiagen, Hilden, Germany). The RNA sequencing (RNA-seq) samples were collected from the cell experiments described above. *β*-actin was used as the reference gene, and no template was used as the negative control. Sequencing files were constructed by Majorbio, sequenced using Illumina HiSeq2000, and annotated against NR, Swiss-Prot, Pfam, COG, GO, and KEGG functional databases.

### 2.8 Real-time quantitative PCR

Total RNA was extracted from the same batch of fibroblasts using TRIzol reagent (Gibco-BRL, Paisley, UK), and RNA concentration and purity were measured using a NanoDrop 2000 (Thermo Scientific of America). For the RT-qPCR analysis, 1 μg total RNA was extracted from cell samples and transformed into cDNA (HiScript Q RT SuperMix for RT-PCR [+gDNA wiper], Vazyme). Five genes were selected based on the RNA-seq results; the target gene and primer sequences for amplification and fluorescence detection are shown in Table 1. *Β*-actin was used as a control gene for all samples. Reaction cycles consisted of denaturation at 95°C for 5 min, followed by 40 cycles of 95°C for 5 s, 58°C for 30 s, and 72°C for 40 s. mRNA levels were quantified using Applied Biosystems sequence detection software and an ABI7500 fluorescence quantitative PCR instrument (LongGene Instruments, Hangzhou China).

**Table 1:** PCR primer sequences of genes used in this study.

Gene name	Primer sequences (5′-3′)	Reverse primer (5′-3′)
*CCL2*	CTTCTGTGCCTGCTGCTC	TGCTGCTGGTGATTCTTCT
*MMP12*	GATCCAAAGGCCGTAATGTTCC	TGAATGCCACGTATGTCATCAG
*IL6*	GAAAGCAGCAAAGAGGCA	CAAATCTGTTCTGGAGGT
*HAS2*	AAGTGCCTTACTGAAACA	GGTAGAAGAGCTGGATTAC
*HBEGF*	CCTCCCACTGTATCCACG	CCTTGCCTTTCTTCTTTCTTTT
*β-actin*	GGCGGCAACACCATGTACCCT	AGGGGCCGGACTCGTCATACT

### 2.9 Statistical analysis

All data are represented as mean ± standard deviation (X ± SD) unless otherwise indicated. Astra software (Version 7.1.2) was used to analyze the polysaccharide molecular weight data. The quantitative analysis of immunofluorescence-stained images was performed using Imagej1.53C (Wayne Rasband, USA) image processing software. RNA-seq data were analyzed using the Majorbio Cloud Platform (www.majorbio.com), and relative changes in target gene expression from PCR were analyzed by the −ΔΔC_T_ method [22].

## 3. Results and discussion

### 3.1 CCE extraction

CCE was obtained from *C*. *cicadae* by hot water extraction, centrifugation, and lyophilization, and the extraction rate was 41.17%. The main active components of CCE were mannitol (10.70%), crude polysaccharides (6.27%), protein (3.57%), and polyphenols (0.38%). Nucleosides and alditols were also detected (data shown in 3.1.3 and 3.1.4), which were indicators of actives that are often of interest to researchers in *C*. *cicadae* studies [7, 23].

#### 3.1.1 CCE polysaccharide composition and average molecular weight

**Fig 1:**
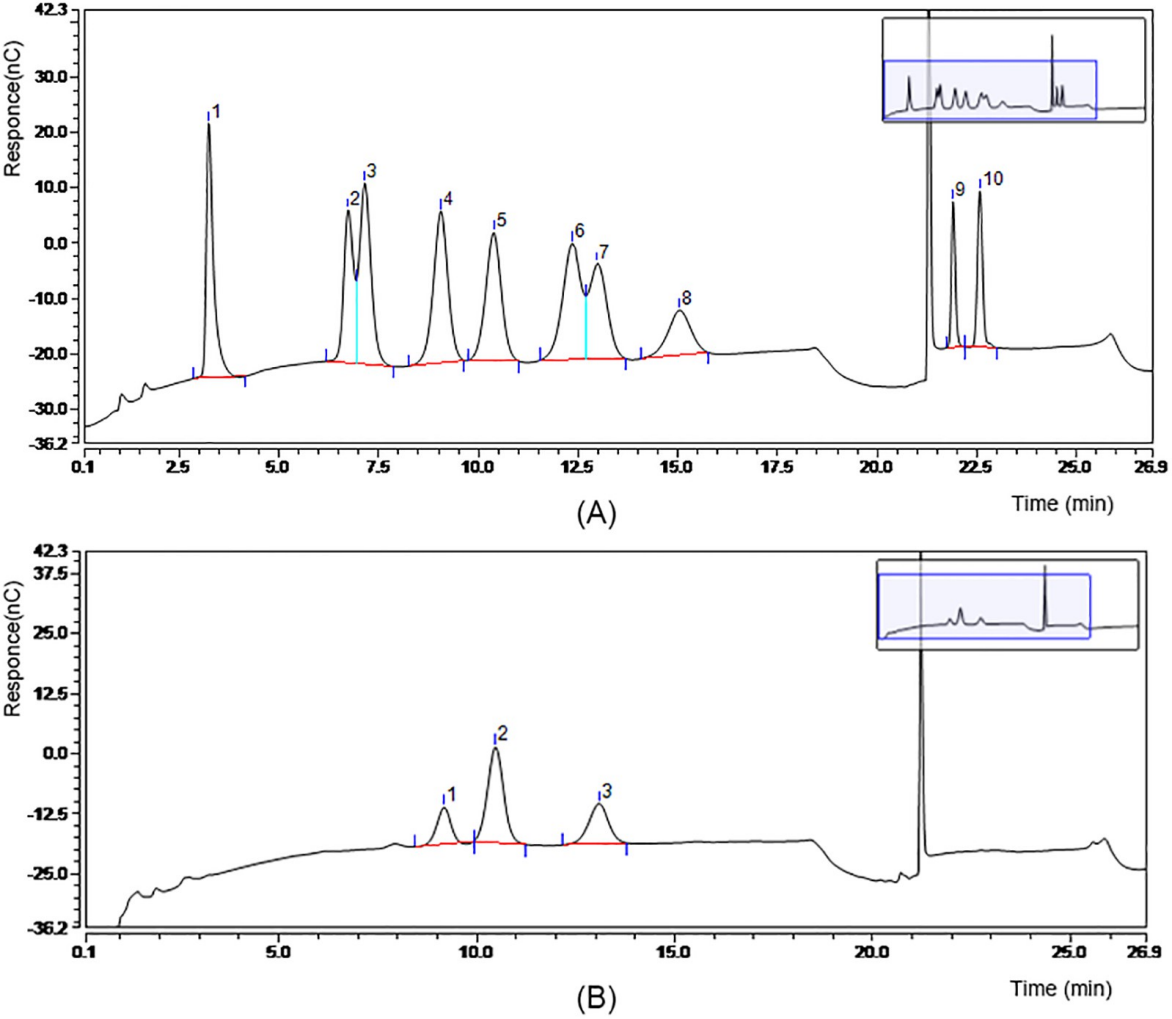
Chromatograph profiles. (A) Monosaccharide composition of standard sample; peaks 1–10: fucose, rhamnose, arabinose, galactose, glucose, xylose, mannose, fructose, glucuronic acid, galacturonic acid. (B) Monosaccharide composition of *C*. *cicadae* extract crude polysaccharides; peaks 1–3: galactose, glucose, mannose.

The average molecular weight and monosaccharide composition of CCE polysaccharides were analyzed. Chromatograms of the mixed standard and CCE are shown in Fig 1A and 1B, respectively. The monosaccharide components of the crude polysaccharides in CCE are galactose, glucose, and mannose (Fig 1B). Glucose was the predominant monosaccharide, and the molar ratio of galactose, glucose, and mannose was 6:22:13, which is consistent with that reported in relevant studies [15, 17, 24]. Previous research has demonstrated that polysaccharides are among the most abundant and bioactive components extracted from *C*. *cicadae* fruiting bodies, cultured mycelium, and fermentation broth [25]. The crude polysaccharides in CCE were composed of three fractions (Table 2), peak 1 (20.27%), peak 2 (18.64%), and peak 3 (61.09%), with average molecular weights of 2.217 × 10^7^, 3.752 × 10^4^ and 2.316 × 10^3^ Da, respectively.

**Table 2:** Average molecular weights of three *C*. *cicadae* extract crude polysaccharide fractions.

Peak	Molecular weight	Relative %
1	2.217 × 10^7^	20.27
2	3.752 × 10^4^	18.64
3	2.316 × 10^3^	61.09

#### 3.1.2 CCE alditols

**Fig 2:**
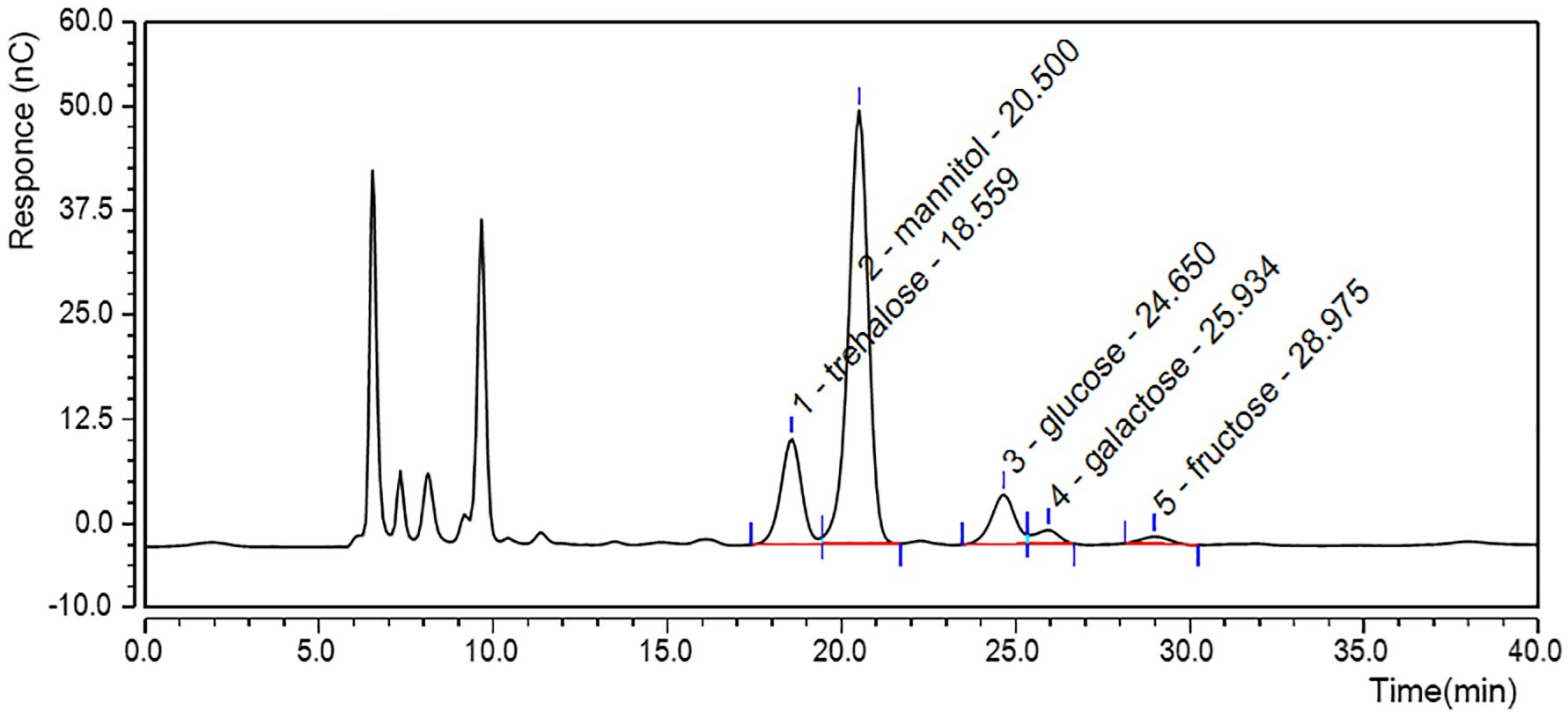
Chromatograph profile of *C*. *cicadae* extract alditols and saccharides.

CCE alditols contents were determined by HPAEC. As shown in Fig 2, five alditols components were detected: 65.25 mg/g trehalose, 107.00 mg/g mannitol, 29.09 mg/g glucose, 13.87 mg/g galactose, and 24.09 mg/g fructose. The mannitol content of CCE is significantly higher than that of *Paecilomyces cicadae* (53.6 mg/g) [26]. Mannitol content is used as an indicator of *C*. *sinensis* quality, and demand for this polyol group is increasing in food and medical fields due to favorable characteristics, such as low metabolism, no glycemic index, non-hygroscopic, anti-oxidative, and natural sweetening properties [27–29]. These attributes reduce consumers’ risk of developing diseases such as diabetes and obesity and can reduce elevated intracranial pressure. Moreover, mannitol in combination with HA can significantly reduce HA degradation [29]. These properties could make CCE an ideal skin care ingredient.

#### 3.1.3 CCE nucleosides

**Fig 3:**
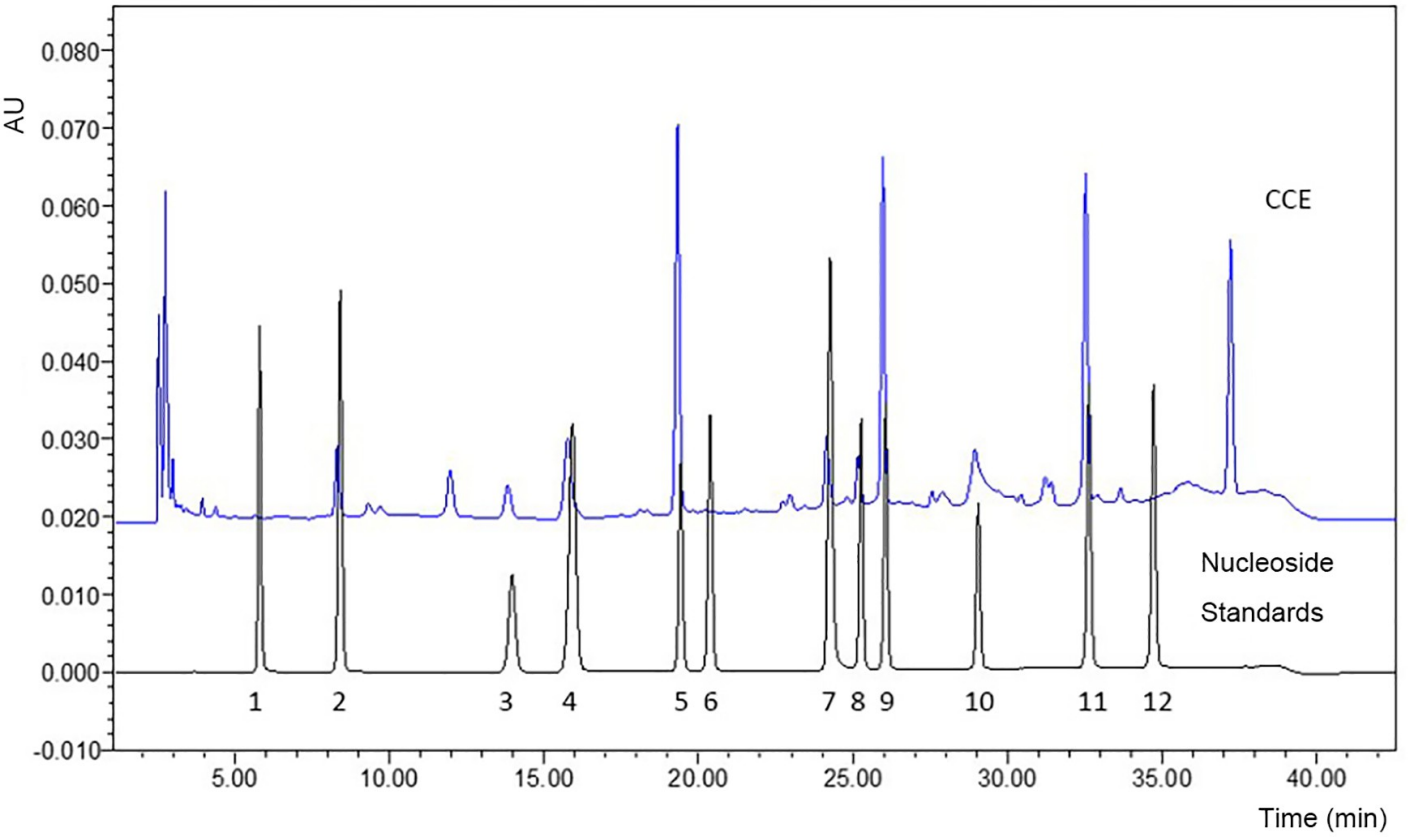
Chromatography profile of nucleoside standards (black signal) and *C*. *cicadae* extract (CCE; blue signal); peaks 1–12: Cytosine, uracil, cytidine, hypoxanthine, uridine, thymine, adenine, inosine, guanosine, thymidine, adenosine, cordycepin.

Nucleosides are one of the main active components of *Cordyceps* fungi [30] and are often used as markers to distinguish *Cordyceps* fungi from knockoffs. Many studies have shown that nucleosides can regulate physiological processes via purine and pyrimidine receptors. Exogenous nucleosides regulate immune function, participate in metabolism, have antibacterial and antiviral effects, and exert therapeutic effects on liver, cardiovascular, and nervous system diseases [31]. Zeng et al. [32] determined the nucleosides of *C*. *cicadae* from different regions, which included uracil, uridine, 2′-deoxyuridine, inosine, guanosine, adenine, thymidine, adenosine, 2’-deoxyadenosine, and cordycepin, with total nucleoside contents ranging from 3.69439 to 8.39772 mg/g [31]. HPLC chromatograms of CCE and mixed standards are shown in Fig 3. Eight nucleosides were detected in CCE, namely uridine, guanosine, adenosine, cytidine, hypoxanthine, inosine, uracil, and adenine, with respective contents of 1.8497, 1.2787, 1.1352, 0.2925, 0.2871, 0.1503, 0.1419, and 0.1237 mg/g. These results were consistent with other reports that uridine content was the highest among nucleosides in *Cordyceps* [32, 33].

### 3.2 CCE antioxidant activities

**Fig 4:**
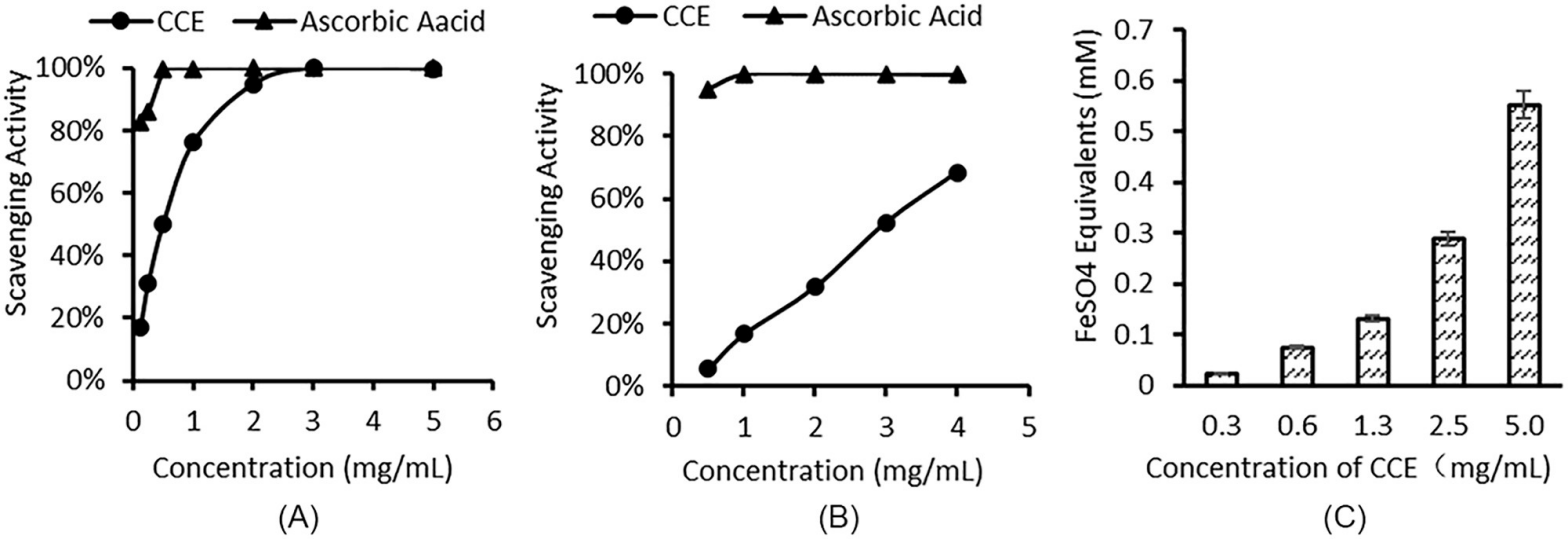
Antioxidant activities of *C*. *cicadae* extract estimated by different methods. (A) ABTS [2,2-azino-bis (3-ethylbenzothiazo-line-6-sulphonic acid)] radical scavenging; (B) DPPH (2,2-diphenyl-1-picrylhydrazil) radical scavenging assay; (C) ferric reducing antioxidant power assay.

The antioxidant activities of CCE were estimated using ABTS (Fig 4A), DPPH (Fig 4B), and FRAP assays (Fig 4C). Using ascorbic acid as a control, the concentration of CCE required to inhibit 50% (IC_50_) of DPPH and ABTS free radical was 4.54 ± 0.10 and 0.36 ± 0.03 mg/mL, respectively. CCE exhibited concentration-dependent ABTS and DPPH scavenging activity; the antioxidant capacity increased with increasing CCE concentrations. Among the antioxidant results of CCE, the IC_50_ value of DPPH was significantly higher than that of ABTS, and CCE at this concentration is already cytotoxic, which may because the ethanol dissolution system of DPPH radical scavenging experiment, which affected the antioxidant effect of aqueous extract CCE. Similarly, the FRAP results showed that the ferric reducing capacity increased in a dose-dependent manner. These results indicate CCE has a strong free radical scavenging capacity.

### 3.3 Cell viability

**Fig 5:**
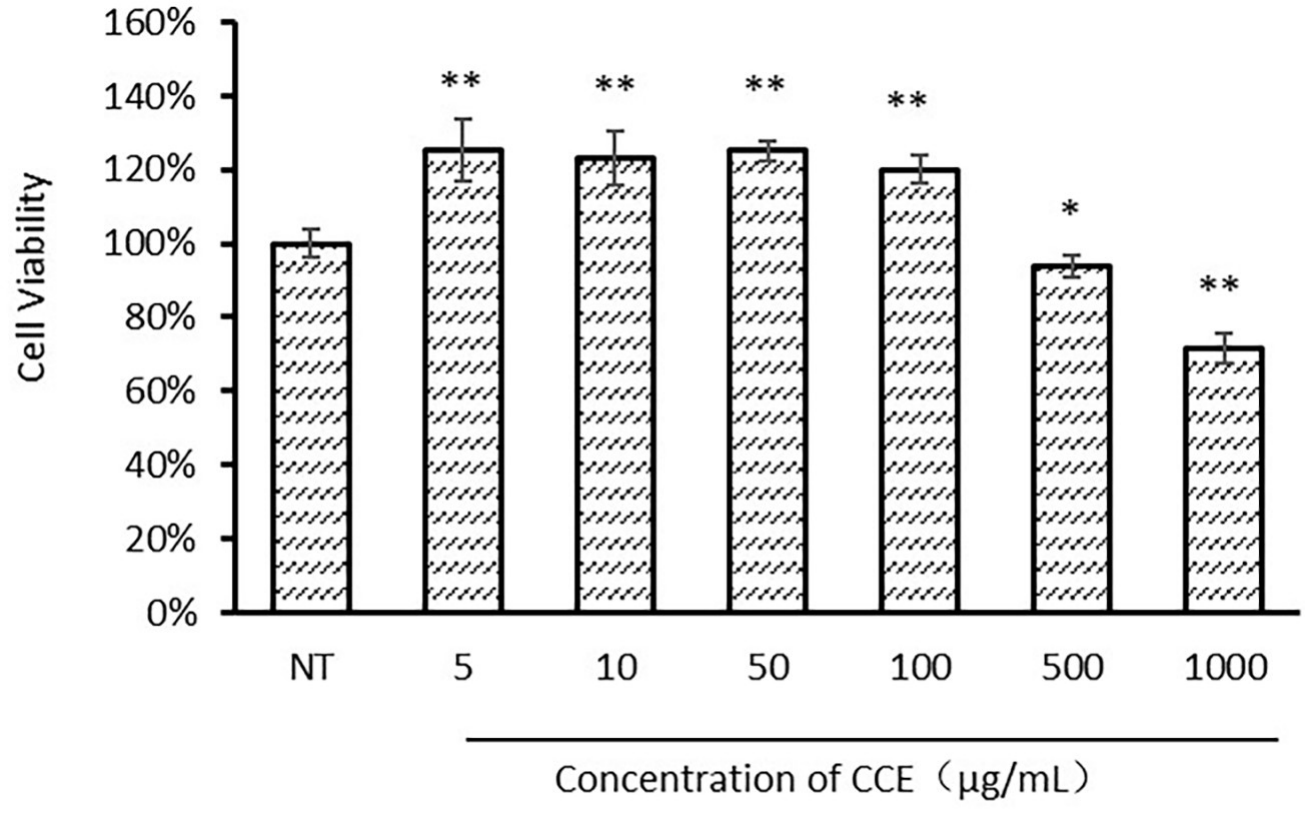
Human skin fibroblasts were cultured with *C*. *cicadae* extract (0, 5, 10, 50, 100, 500 and 1000 μg/mL) for 24 h, and cell viability was measured by MTT assay (*n* = 3), * *P* < 0.05, ** *P* < 0.01 compared with the control group.

The cytotoxicity of CCE was assessed using an MTT assay of cultured human skin fibroblasts treated with various concentrations of CCE. As shown in Fig 5, CCE enhanced cellular viability at concentrations of 5 (*p* = 6.04×10^−5^), 10 (*p* = 6.37×10^−5^), 50 (*p* = 1.34×10^−7^) and 100 μg/mL (*p* = 4.43×10^−6^), and cell viability values remained 94% at concentrations up to 500 μg/mL (*p* = 0.01) and possessed cytotoxicity at 1000 μg/mL (*p* = 2.45×10^−7^). CCE did not reduce fibroblast cell viability at concentrations ranging from 5 to 100 μg/mL and was slightly toxic to fibroblasts at concentrations above 500 μg/mL.

### 3.4 CCE promotes HA synthesis in human fibroblasts

**Fig 6:**
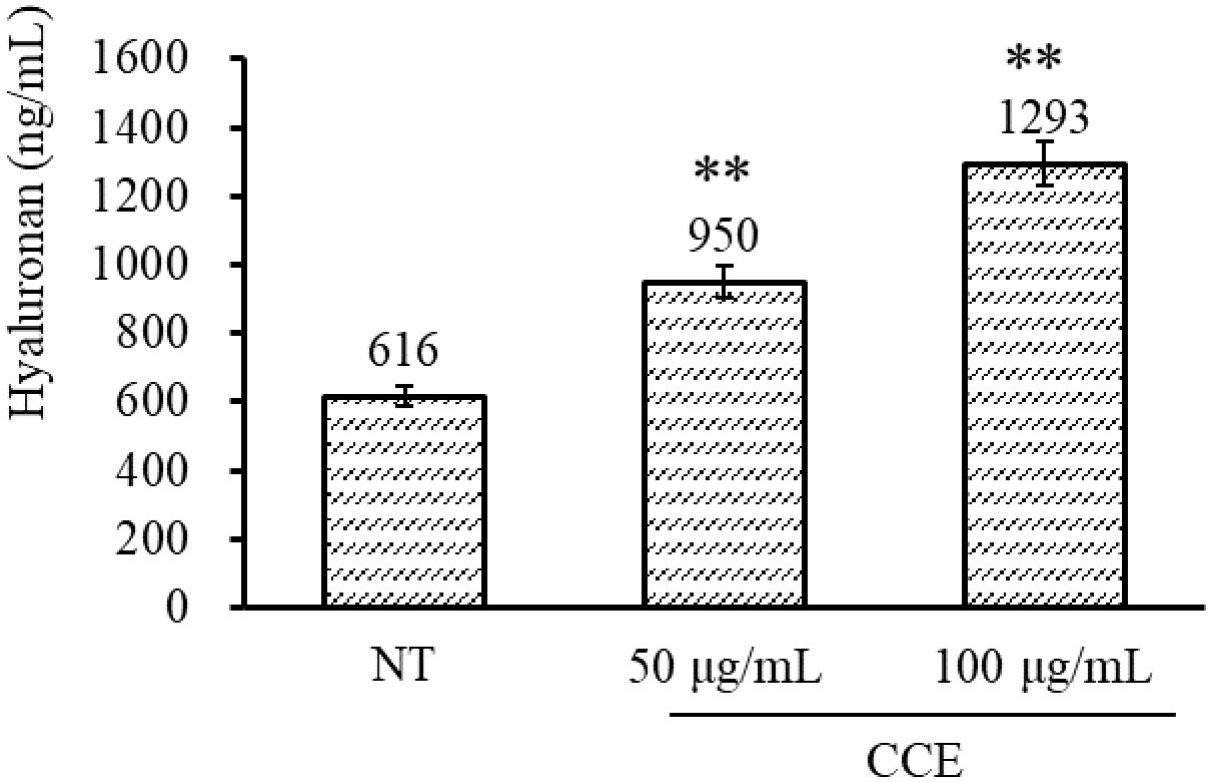
The content of hyaluronan of fibroblasts in the CCE 50, 100 μg/mL treated and non-treatment group. * *P* < 0.05, ** *P* < 0.01 compared with the control group.

Skin aging is characterized by uneven pigmentation, impaired skin elasticity and strength, and rough texture. Skin integrity is maintained via the ECM, in which HA plays an important role. Many studies have demonstrated the effects of natural extracts on skin [34]. The hyaluronic acid content in fibroblasts cells was determined using ELISA kits. As shown in Fig 6, treatment of fibroblast cells with 50 μg/mL and 100 μg/mL CCE enhances the HA content to 950 ± 52 and 1293 ± 142 ng/mL respectively, which is significantly more than that in the non-treatment (NT) group (p = 0.0067). This result demonstrated CCE could promote HA synthesis in skin fibroblasts. And the promotion of HA synthesis induced by CCE might be related to its flavonoid content [35].

### 3.5 Immunofluorescence staining analysis of HA expression

**Fig 7:**
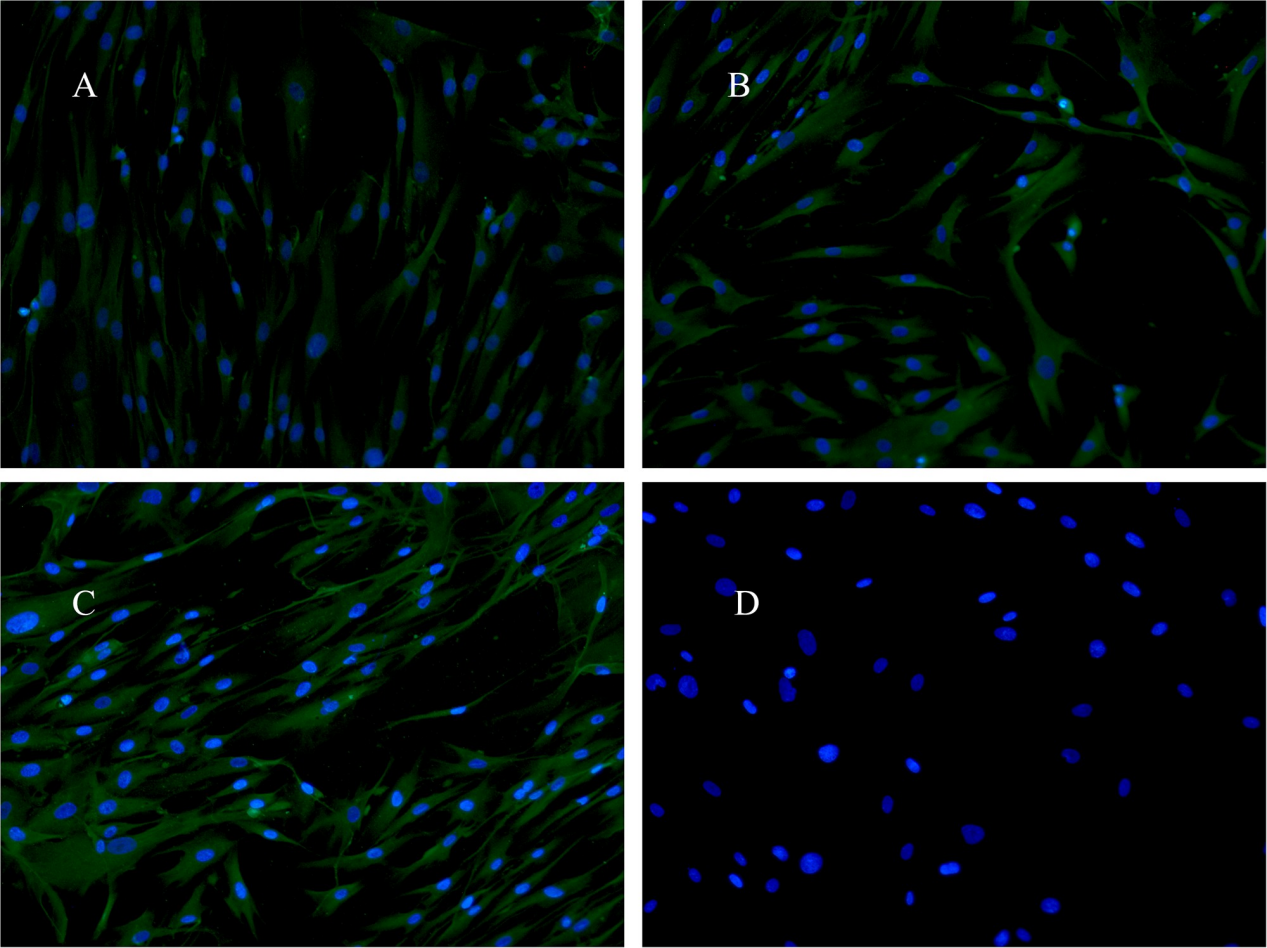
Immunofluorescence staining of fibroblast nuclei (blue) and hyaluronan (green) at 10× magnification. Fibroblasts were non-treated (A), treated with *C*. *cicadae* extract at concentrations of 50 μg/mL (B) and 100 μg/mL (C), treated without the antibody 488 as negative control (D).

Using immunofluorescence staining of fibroblasts treated with CCE to further reveal the effects on HA expression. Under the fluorescence microscope, the nucleus was blue, and HA was green. Compared to the NT group (Fig 7A), 50 μg/mL CCE (Fig 7B) and 100 μg/mL CCE (Fig 7C) increased HA expression, and the negative control (Fig 7D) showed that there were no false positives.

### 3.6 Transcriptional analysis

To determine the effects of CCE on HA synthesis in fibroblasts, we quantified HA-related gene expression in cell cultures treated with 50 μg/mL CCE.

#### 3.6.1 Transcriptional response

In the CCE group, 1.48 × 10^8^ reads were obtained by sequencing, and 1.47 × 10^8^ clean reads were obtained by filtration, accounting for 99.18% of the total reads. In the NT group, 1.57 × 10^8^ reads were obtained by sequencing, and 1.56 × 10^8^ clean reads were obtained by filtration, accounting for 99.21% of the total reads. The percentage of clean reads obtained by each sample was greater than 99%, and the sequencing quality was high, meeting the information analysis requirements.

#### 3.6.2 Differentially expressed gene (DEG) analysis

The transcriptional regulation of CCE-treated (50 μg/mL) and NT fibroblasts was analyzed by RNA-seq. A total of 1192 significant DEGs (up or downregulated > 1.2) were detected between CCE-treated and NT fibroblasts, of which 417 genes were upregulated, and 775 genes were downregulated. DEGs were selected based on *P*-values generated by the Benjamini-Hochberg correction using a uniform threshold of *P* < 0.05.

**Fig 8:**
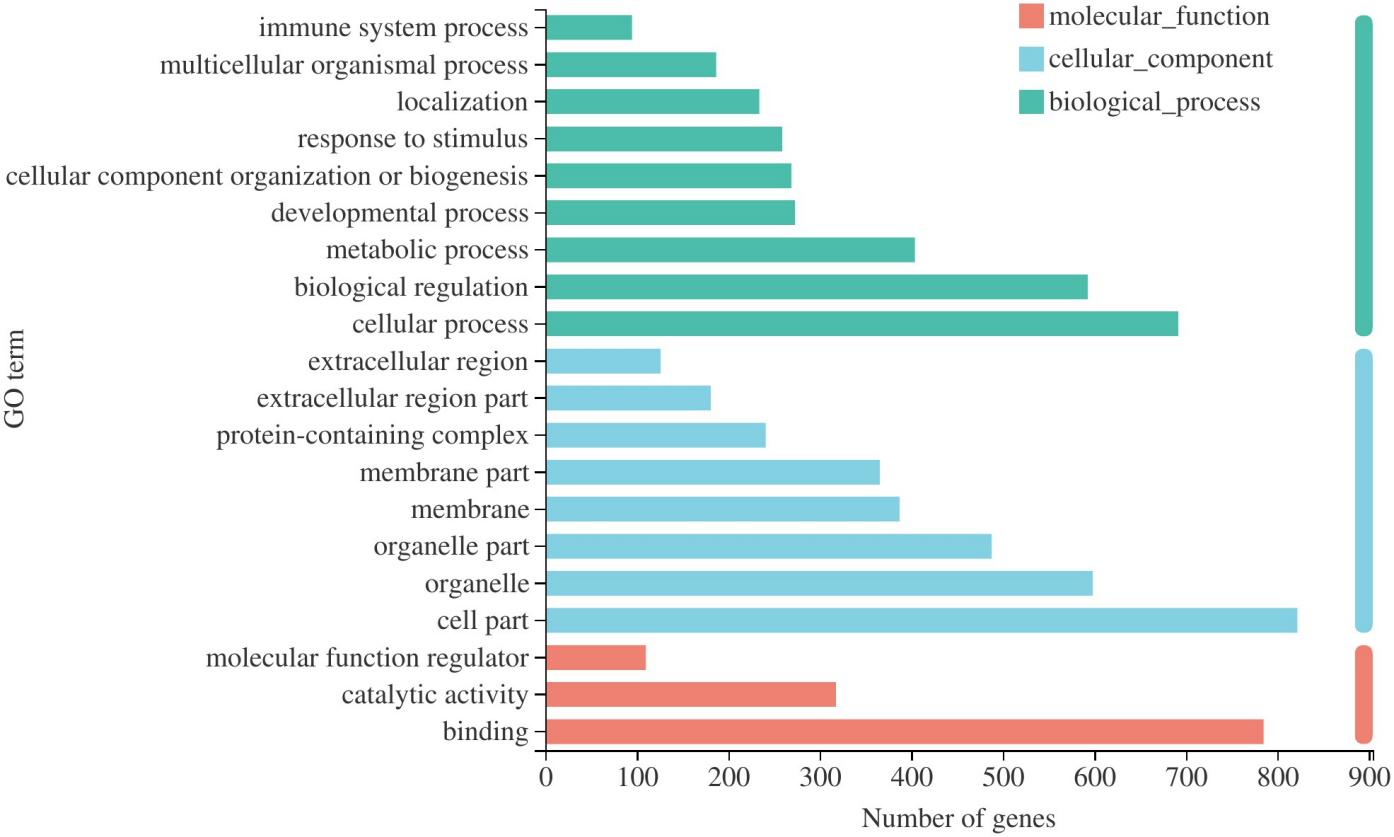
Gene ontology analysis of differentially expressed genes between *C*. *cicadae* extract-treated (50 μg/mL) and non-treated fibroblasts.

The GO analysis detected 20 significantly enriched terms (Fig 8). GO terms are divided into biological processes, cell components, and molecular function categories. The most significantly enriched GO terms were involved in biological processes, namely, cellular processes, biological regulation, and metabolic processes.

**Fig 9:**
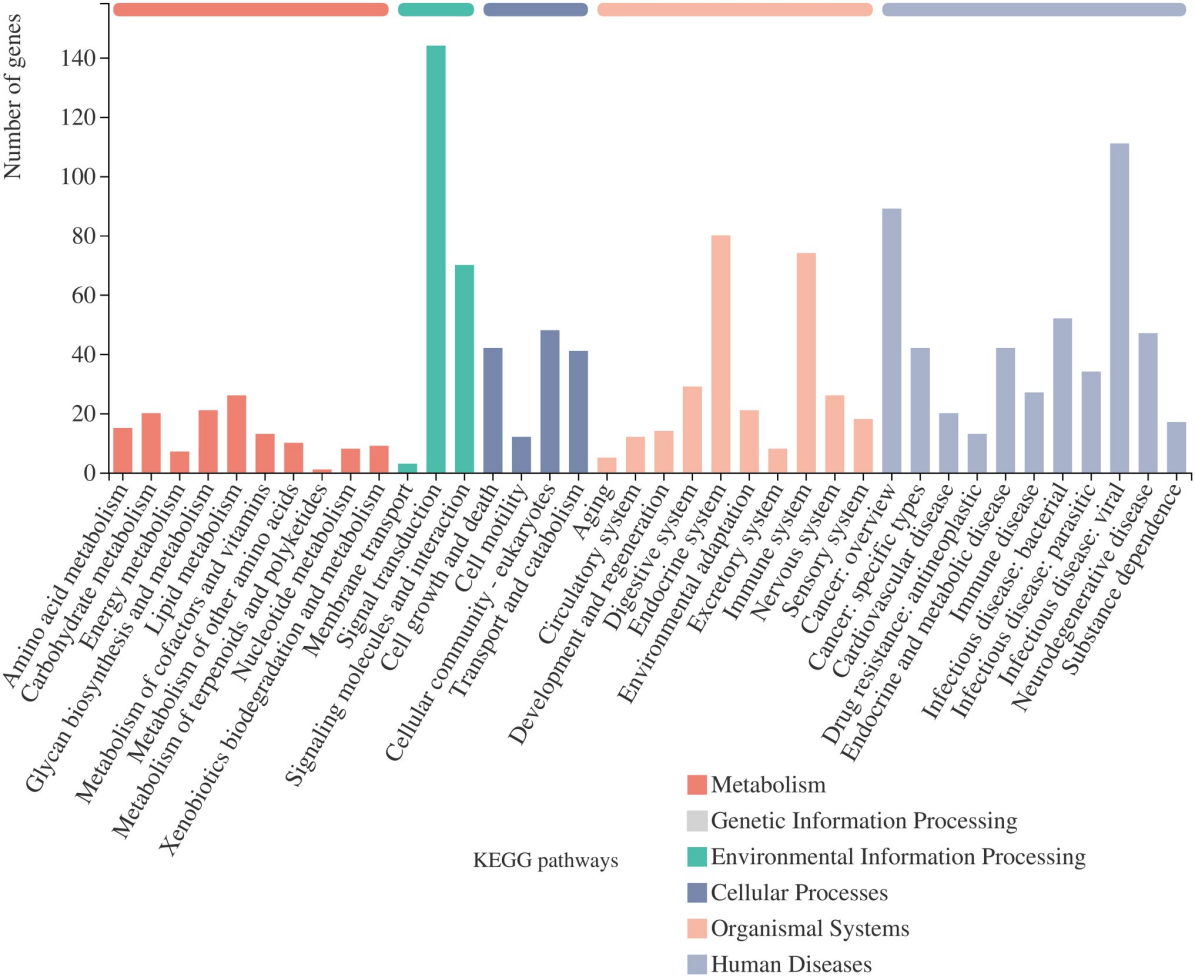
Distribution of enriched KEGG (Kyoto Encyclopedia of Genes and Genomes) pathways of differentially expressed genes between *C*. *cicadae* extract-treated (50 μg/mL) and non-treated fibroblasts.

According to the KEGG metabolic pathway analysis (Fig 9), DEGs were mostly enriched in pathways related to immune diseases and signal transduction. Total KEGG enrichment showed that viral protein interaction with cytokine and cytokine receptor, cytokine-cytokine receptor interaction, AGE-RAGE signaling pathway in diabetic complications, protein digestion and absorption, TNF signaling pathway, ECM-receptor interaction, and PI3K-Akt signaling pathway had a high significance and number distribution.

HA is an intercellular matrix component. Of the genes upregulated in the CCE-treated group compared with the NT group, nine of them were associated with HA metabolism, intercellular matrix metabolism, and fibroblast proliferation and differentiation (Table 3). The effects of CCE on skin fibroblasts may be attributed to the regulation of these genes.

**Table 3:** Differentially expressed genes related to hyaluronan synthesis between *C*. *cicadae* extract-treated and non-treated fibroblasts.

Gene_id	Gene name	Gene description	Log2 (fold change)	*P*-value	*P*-adjusted	Regulation
ENSG00000123610	*TNFAIP6*	TNF alpha-induced protein 6	0.428	0.018	0.331	up
ENSG00000170961	*HAS2*	Hyaluronan synthase 2	0.277	< 0.001	< 0.001	up
ENSG00000262406	*MMP12*	Matrix metallopeptidase 12	-0.473	0.050	0.535	down
ENSG00000136244	*IL6*	Interleukin 6	0.668	< 0.001	< 0.001	up
ENSG00000108691	*CCL2*	C-C motif chemokine ligand 2	0.571	< 0.001	< 0.001	up
ENSG00000113070	*HBEGF*	Heparin-binding EGF-like growth factor	0.489	0.001	0.056	up
ENSG00000259753	*AC068234*.*1*	Novel protein	1.604	< 0.001	< 0.001	up
ENSG00000134070	*IRAK2*	Interleukin 1 receptor-associated kinase 2	0.368	0.030	0.426	up
ENSG00000109321	*AREG*	amphiregulin	3.250	0.020	0.352	up

The genes of interest were searched in GO and KEGG, and some of these genes (e.g., *IL6* and *CCL2*) involved in cell proliferation and intercellular matrix assembly were related to immune regulation. The results of GO enrichment and KEGG enrichment analyses (Figs 8 and 9) also showed that most terms were related to inflammatory response and cytokine activity. Thus, CCE may play a role in immune regulation, which can be further explored in the future; however, the DEGs also have other known functions. The *TNFAIP6*, *IL6*, *CCL2*, *HBEGF*, *IRAK2*, and *AREG* genes are associated with the mitogen-activated protein kinase (MAPK) pathway, which transmits regulatory signals to the nucleus and is related to fibroblast proliferation and division. The MAPK pathway includes ERK/P-ERK, P38/P-P38, and JNK/P-JNK [36]. The extracellular-regulated protein kinase (ERK) pathway is mainly involved in cell proliferation, differentiation, migration, survival, and apoptosis [37]. The ERK pathway promotes HA synthesis when activated by appropriate external stimuli such as photodynamic therapy [36]. According to the GO analysis, the upregulation of CCL2 positively regulates the ERK1 and ERK2 cascade.

The upregulated gene hyaluronan synthase 2 (*HAS2*) encodes one of the isoenzymes that catalyze the addition of N-acetyl-glucosamine and glucuronic acid monosaccharides to the newly formed HA polymer [38]. *HAS2* is the major HA synthase in dermal fibroblasts [39], and it specializes in the synthesis of high polymer mass HA. Matrix metalloproteinases (MMPs) are a family of zinc-dependent endopeptidases involved in physiological processes, such as cell proliferation and apoptosis, tissue remodeling, hematopoietic regulation, wound repair, and angiogenesis [40]. In terms of the effect of CCE on the ECM, *MMP12* was downregulated in CCE-treated fibroblasts. Previous studies have shown that the ECM is continuously lost during externally induced skin aging, which is often associated with MMP upregulation [8]. Thus, CCE inhibited the gene associated with ECM catabolic processes.

Some studies have shown that polysaccharides derived from *Lactobacillus plantarum* downregulate MMPs to prevent skin aging and ECM loss, the molecular weights of those polysaccharides ranged from 221 to 79,967 Da [41]. The polysaccharides extracted from CCE were 20.27% macromolecular polysaccharides and 79.73% small molecular weight polysaccharides (2,316 to 37,520 Da), which is on the order of magnitude indicated in Lee’s study. Lee et al. [41] also found that the anti-aging polysaccharide was composed of ribose, glucose, and mannose in a ratio of 4.0:1.5:1.0 [8]. CCE contains glucose and mannose, which is similar to Lee’s study. The effect of CCE on HA synthesis in fibroblasts may involve other regulatory pathways, and future studies on HA synthesis are warranted.

### 3.7 RT-qPCR verification

**Fig 10:**
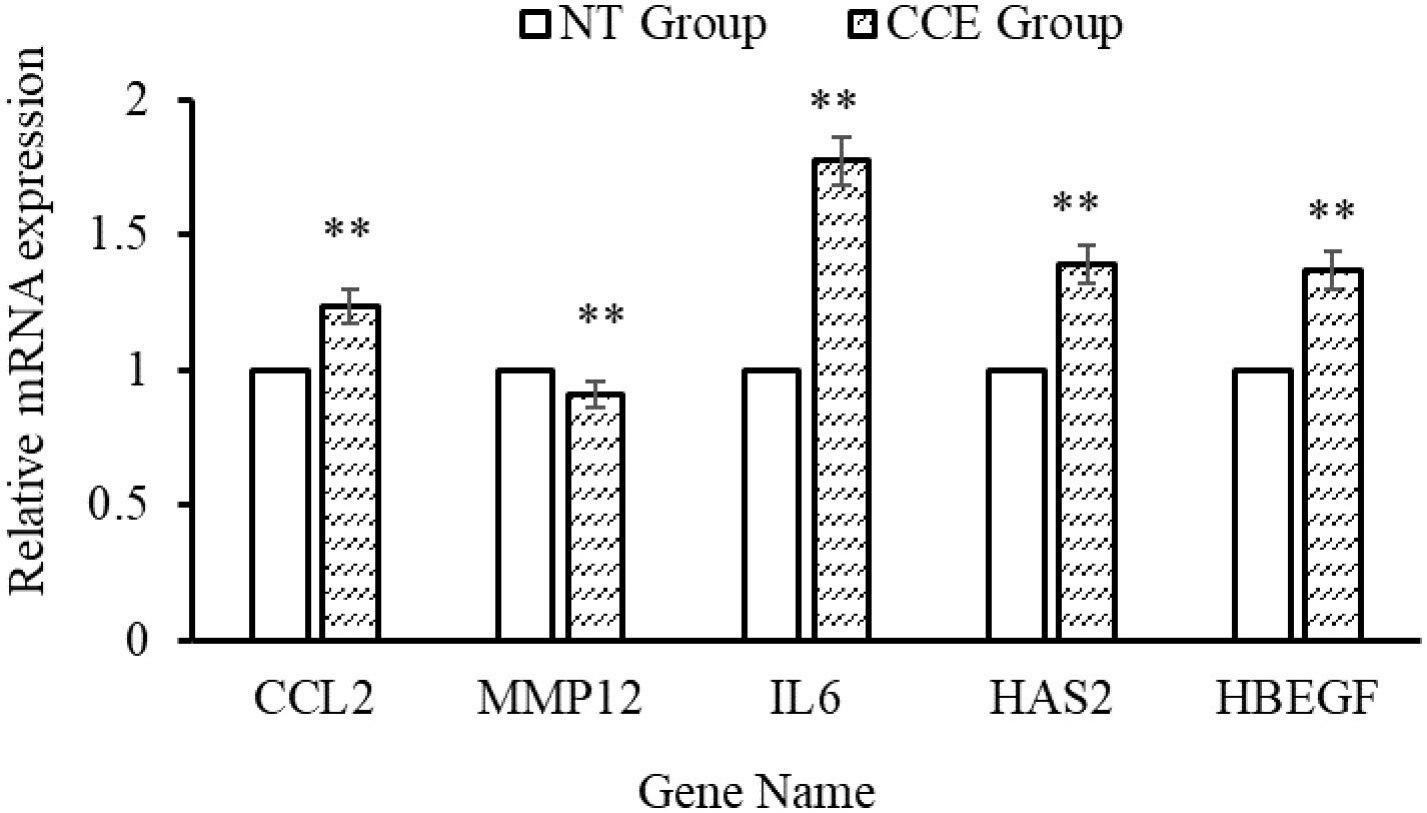
RT-qPCR expression levels of five hyaluronan-related genes in *C*. *cicadae* extract (CCE)-treated and non-treated (NT) fibroblasts, * *P* < 0.05, ** *P* < 0.01 compared with the control group.

To further verify HA-related gene expression, five selected genes (*CCL2*, *MMP12*, *IL-6*, *HAS2*, and *HBEGF*) were amplified and quantified using RT-qPCR. Compared with the NT group, *CCL2* (*p* = 0.0017), *IL-6* (*p* = 3.02×10^−5^), *HAS2* (*p* = 0.0002), and *HBEGF* (*p* = 0.006) gene expression was higher and *MMP12* (*p* = 0.0002) expression was lower in the CCE-treated group (Fig 10). CCE-treated fibroblasts exhibited upregulated expression of *HAS2*, which is involved in synthesizing large HA molecules. Genes such as epidermal growth factor-related gene *HBEGF*, *CCL2*, and other genes related to fibroblast differentiation and proliferation were also upregulated, whereas *MMP12* was significantly downregulated in fibroblasts treated with CCE. The results were consistent with the transcriptome data, confirming the reliability of the RNA-seq.

## 4. Conclusion

CCE promoted HA synthesis in human skin fibroblasts. Furthermore, RNA-seq analysis and RT-qPCR verification demonstrated that CCE promoted the expression of *HAS2*, epidermal growth factor-related genes, *HBEGF*, *CCL2*, *IRAK2*, and genes related to fibroblast differentiation and proliferation, and significantly downregulated the expression of *MMP12*. The results of this study indicate that CCE has strong antioxidant activity, which may be related to its composition of eight nucleosides, six alditols, active polysaccharides, protein, and polyphenols. Based on the traditional role of *C*. *cicadae* as antioxidant and anti-aging medicine, we studied the antioxidant activity and HA synthesis-promoting effects. Thus, CCE can be used as a potential moisturizer and anti-aging agent in functional foods and cosmetics in the future.

## Abbreviations

ABTS: 2,2-azino-bis (3-ethylbenzothiazo-line-6-sulphonic acid)
AGE-RAGE: receptor for advanced glycation end products
BSA: bovine serum albumin
CCE: *Cordyceps cicadae* (Miq) extract
COG: clusters of orthologous groups
DEGs: differentially expressed genes
DMEM: Dulbecco’s modified eagle medium
DPBS: Dulbecco’s phosphate-buffered saline
DPPH: 2,2-diphenyl-1-picrylhydrazil
ECM: extracellular matrix
ERK/P-ERK: extracellular regulated protein kinases/phosphor- extracellular regulated protein kinases
FRAP: ferric reducing antioxidant power
GO: Gene Ontology
HA: hyaluronan
HAS2: HA synthase 2
HPAEC: high-performance anion-exchange chromatography
HPLC: high-performance liquid chromatography
JNK/PJNK: c-Jun N-terminal kinase/phosphor- c-Jun N-terminal kinase
KEGG: Kyoto Encyclopedia of Genes and Genomes
MMP: matrix metallopeptidase
MTT: 3-(4,5-dimethylthiazol-2-yl)-2,5-diphenyl-2H-tetrazolium bromide
NR: non-redundant protein sequence database
NT group: non-treatment groupA
Pfam: protein families database
PI3K-Akt: phosphoinositide 3-kinase
RNA-seq: RNA sequencing
RT-qPCR: real-time quantitative polymerase chain reaction
Swiss-prot: Swiss-Prot protein sequence database
TCM: traditional Chinese medicine
TNF: tumor necrosis factor
TPTZ: 2, 4, 6-tripyridyl-s-triazine
